# Combination Compositions Composed of l-Glutamine and Si-Jun-Zi-Tang Might Be a Preferable Choice for 5-Fluorouracil-Induced Intestinal Mucositis: An Exploration in a Mouse Model

**DOI:** 10.3389/fphar.2020.00918

**Published:** 2020-06-17

**Authors:** Liping Qu, Wanxian Tan, Jing Yang, Limin Lai, Sili Liu, Jianming Wu, Wenjun Zou

**Affiliations:** ^1^School of Pharmacy, Chengdu University of Traditional Chinese Medicine, Chengdu, China; ^2^School of Pharmacy, Southwest Medical University, Luzhou, China

**Keywords:** combination compositions, l-glutamine, Si-Jun-Zi-Tang, 5-fluorouracil, intestinal mucositis

## Abstract

Intestinal mucositis is a common toxicity of many anti-neoplastic therapies that negatively influences health, the quality of life, economic outcomes, and even the success of cancer treatment. Unfortunately, there is presently no optimal medicine that is able to effectively manage this condition. l-glutamine is one of the most frequently used agent in practice among the limited treatment choices due to its safety and inexpensiveness despite there being a lack of evidence. Previous studies indicated that l-glutamine may alleviate mucositis and mucosal atrophy but failed to improve patients' macroscopic conditions, such as the occurrence of diarrhea. A compound glutamine capsule (G-SJZ), composed of l-glutamine and the traditional Chinese herbal formula Si-Jun-Zi-Tang, has been used in China for 23 years to treat many types of gastrointestinal diseases, including gastrointestinal reactions induced by radiotherapy and chemotherapy. However, the exact effect of G-SJZ on intestinal mucositis is unclear, and moreover, whether l-glutamine combined with Si-Jun-Zi-Tang is more effective than l-glutamine alone have not been studied. In the current study, we explored the effects of G-SJZ and l-glutamine in a mouse model of intestinal mucositis induced by 5-fluorouracil (5-Fu). The results revealed that pretreatment with G-SJZ ameliorated the physical manifestations of weight loss and the severity of diarrhea following continuous 5-Fu injections in mice. Likewise, the histopathological damage and the destruction of villus and crypt structures in the intestinal mucosa as well as the increase in circulating intestinal injury markers caused by 5-Fu were reversed with G-SJZ pretreatment. Furthermore, the protective effect of G-SJZ was accompanied by modulations in the immunohistochemical expression of tight junction proteins. Interestingly, although treatment with a dose of l-glutamine alone that was equivalent to the dose in G-SJZ also showed a protective effect, it did not appear to be as strong as treatment with G-SJZ. Si-Jun-Zi-Tang in G-SJZ may compensate for the deficiencies of l-glutamine in this model which seems not to be related to the regulation of tight junction proteins. Our study is the first to suggest that the combined use of l-glutamine and Si-Jun-Zi-Tang might be more effective than l-glutamine alone despite exact mechanism still needs further study. Because of the limited number of therapeutic agents, G-SJZ is likely to be a preferable choice for intestinal mucositis.

## Introduction

Most non-surgical anti-neoplastic therapies produce anticancer effects *via* their potential to inhibit the division of rapidly dividing cancer cells. Because epithelial cells of the gastrointestinal tract are also rapidly dividing, many anticancer drugs have a high tendency to damage these cells while targeting cancer cells. As a consequence, intestinal mucositis, which is frequently associated with diarrhea and is characterized by the inflammation and ulceration of intestinal mucosa, becomes one of the most common adverse effects following anti-neoplastic treatments (Gibson and Stringer, 2009; Bowen et al., 2019). As an unwanted toxicity, intestinal mucositis not only occurs in treatment with traditional chemotherapies, e.g., 5-fluorouracil (5-FU) and irinotecan, but also occurs with targeted therapies, such as tyrosine-kinase inhibitors and epidermal-growth-factor inhibitors, which has also posed challenges in clinical practice, given the increasing exposure of cancer patients to these agents (Pusztaszeri et al., 2007; Takeda et al., 2015; Van Sebille et al., 2015). Although the absolute occurrence is uncertain, several randomized clinical studies reported grade 3-4 severe diarrhea associated with intestinal mucositis in 5% to 47% of patients who received diverse anticancer regimens, including FOLFOXIRI, irinotecan, FOLFIRI with cetuximab, FLOX with cetuximab, etc. (Andreyev et al., 2014). Erlotinib was reported to induce all grades of diarrhea in 67.9% of patients with advanced non–small-cell lung cancer in a phase III trial, with 12.4% of patients suffering from grade 3 diarrhea (Herbst et al., 2005). Immune checkpoint inhibitors, a novel category of cancer treatment, may also affect the gastrointestinal tract, leading to severe diarrhea (Kuo et al., 2018). The effective prevention and treatment of intestinal mucositis have become important issues in the clinical practice of oncologic treatment.

As an important non-essential amino acid in the gastrointestinal tract, l-glutamine acts not only as the major energy source but also as a necessary substrate for nucleotide synthesis in epithelial cells and other rapidly proliferating cells without stimulating tumor growth (Klimberg et al., 1990; Klimberg and McClellan, 1996). Hence, in addition to the roles in diseases with significant metabolic stress, e.g., sepsis and multiple trauma, l-glutamine treatment has been suggested and extended to mucositis induced by chemotherapy and radiotherapy due to its supportive role in the trophism of the intestinal epithelium (Skubitz and Anderson, 1996; García-de-Lorenzo et al., 2003; Savarese et al., 2003; Beutheu et al., 2014). However, l-glutamine failed to be listed in the guidelines for the management of cancer treatment-related gastrointestinal mucositis released by the Multinational Association of Supportive Care in Cancer/International Society for Oral Oncology (MASCC/ISOO) because there is limited high-level evidence or because inconsistent results have been shown in clinical studies (Gibson et al., 2013; Lalla et al., 2014; Bowen et al., 2019). Early studies also indicated that l-glutamine did not reduce the occurrence of chemotherapy-induced diarrhea or improve the nutritional status and food intake in cancer patients, even though it relieved mucositis and mucosal atrophy (Bozzetti et al., 1997; Decker-Baumann et al., 1999).

Interestingly, in China, there is a compound product whose active ingredients include l-glutamine and Si-Jun-Zi-Tang, a traditional Chinese herbal formula composed of *Ginseng* radix et rhizoma, *Atractylodis macrocephalae* rhizoma, *Poria* and *Glycyrrhizae* radix et rhizoma. The combination composition (G-SJZ) was approved as a compound glutamine capsule 23 years ago and has been widely used in gastrointestinal diseases such as ulcerative colitis (Dai et al., 2017), irritable bowel syndrome (Li, 2018) and gastrointestinal reactions induced by radiotherapy and chemotherapy (Han et al., 2017; Li and Liang, 2018). However, the exact effect of G-SJZ on intestinal mucositis has not yet been studied. Exposure of mice to 5-FU, whose most common side effects include intestinal mucositis, may mimic the intestinal damage that occurs in cancer patients (Andreyev et al., 2014). Hence, in the present study, we used this model to examine the protective effects of G-SJZ on intestinal mucositis induced by 5-Fu. Moreover, whether the combination of Si-Jun-Zi-Tang and l-glutamine was more effective than l-glutamine in mice was also examined.

## Materials and Methods

### Materials and Reagents

The combination G-SJZ composition was purchased from Diao Group Chengdu Pharmaceutical Co., Ltd. (Chengdu, China; batch number, 1804022). This preparation was composed of l-glutamine (C_5_H_10_N_2_O_3_, Purity≥99.8%) provided by Kyowa Hakko Kogyo Co., Ltd. (Kyoto, Japan) and traditional Chinese herbal ingredients, including *Ginseng* radix et rhizome (*Panax ginseng* C. A. Mey.), *Atractylodis macrocephalae* rhizome (*Atractylodes macrocephala* Koidz.), *Poria (Poria cocos* (Schw.) Wolf.) and *Glycyrrhizae* radix et rhizome (*Glycyrrhiza uralensis* Fisch.). According to the drug standard (WS-10001-(HD-0836)-2002) issued by Sichuan institute for drug control, each 1 g of G-SJZ contained 0.6 g of l-glutamine and 0.4 g of aqueous extract which refers to a total of 1 g of the four raw herbal materials (Sichuan Chinese Herb Slices Co., Ltd., Chengdu, China), including 0.25 *g* of *Ginseng* radix et rhizome, 0.25 g of *Atractylodis macrocephalae* rhizome, 0.25 g of *Poria*, and 0.25 g of *Glycyrrhizae* radix et rhizome. 5-FU was purchased from Sigma-Aldrich (St. Louis, USA). ELISA kits for d-lactic acid and endotoxin were purchased from Nanjing Jiancheng Bioengineering Institute (Nanjing, China). An ELISA kit for diamine oxidase (DAO) was purchased from Elabscience Biotechnology Co., Ltd. (Wuhan, China). Goat anti-rabbit IgG-HRP and rabbit anti-ZO-1 antibodies were purchased from Gene Tex Inc. (California, USA). Rabbit anti-occludin and rabbit anti-claudin-1 antibodies were purchased from Abcam Inc. (Cambridge, UK).

### Animals

Male ICR mice weighing 18-22 g were purchased from Vital River Laboratory Animal Technology Co., Ltd. (Beijing, China). The animals were housed with controlled temperatures (25 ± 2°C), constant humidity (50 ± 10%) and a 12-h light/12-h dark cycle. Both standard laboratory food and water were available to the mice *ad libitum*. All the mice were housed in standard cages with 6 mice per cage, which were cleaned every day. This study was approved by the animal ethics committee of Chengdu University of Traditional Chinese Medicine, China. All procedures were carried out in accordance with the Guiding Principles for the Care and Use of Laboratory Animals of China.

### UHPLC-ESI-MS/MS Analysis of G-SJZ

Ultra-high performance liquid chromatography (UHPLC, Hadano, Japan) system equipped with a 8045 electrospray ionization tandem mass spectroscopic (ESI-MS/MS) was used to identify the characteristic components in G-SJZ. At first, 14.6 mg l-glutamine was dissolved in 10-ml ultrapure water and the 1.46-mg/ml l-glutamine solution was prepared for the further analysis. Then 0.23 g of G-SJZ was ultrasonically dissolved in 2.3 ml ultrapure water (100 mg/ml), and the cooled solution was centrifuged at 15,000 rpm for 20 min. After that, the supernatant was transferred into a 1.5-ml glass bottle, and 10 μl of the solution was injected into the UHPLC system for the chemical analysis. The chromatographic separation was carried out on a XCharge C_18_ column (150 mm × 2.1 mm, 5 μm) at a flow rate of 0.3 ml/min. The elution system consisting of mobile phase A (0.1% formic acid) and mobile phase B (acetonitrile) was set as follows: 0 to 15 min, 2% to 80% (B). The UV-vis detection wavelength was set at 248 nm. For the optimized conditions of mass spectrum in negative and positive ion modes, ion spray voltage was 3.5 kV, interface temperature was 400°C and the flow rate of nebulizing gas was set at 20 L/min. Data were acquired in scan mode from *m*/*z* 100-2000 Da with 2.0 spectra/s. Data analysis was carried out using Shimadzu LabSolutions software B.01.03 (Hadano, Japan).

### Experimental Design

**Figure 1:**
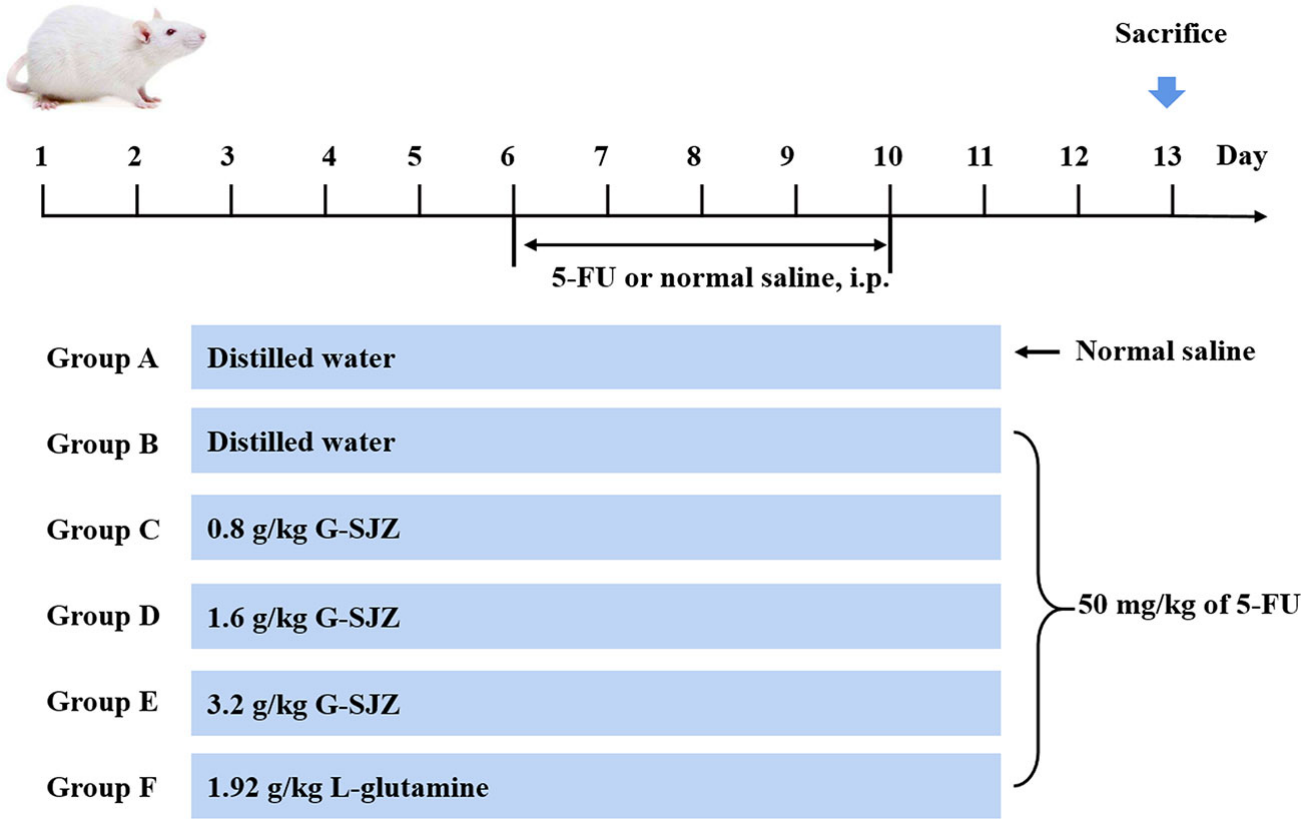
Experimental design. ICR mice were divided into six groups (n = 12 per group) and treated with stilled water or corresponding drug solution for 13 days. From day 6 to day 10 mice were subjected normal saline or 50 mg/kg of 5-FU injection. Animals were sacrificed on the 13^th^ day. G-SJZ, combination composition composed of glutamine and Si-Jun-Zi-Tang; Gln, glutamine.

Following 1 week of acclimation, 72 mice were randomly divided into six groups (n = 12 per group): a normal control group (A), a model group (B), a 0.8 g/kg G-SJZ-treated group (C), a 1.6 g/kg G-SJZ-treated group (D), a 3.2 g/kg G-SJZ-treated group (E) and a 1.92 g/kg l-glutamine-treated group (F). Mice in groups C, D, and E were given 0.8, 1.6, and 3.2 mg/kg G-SJZ orally, and mice in group F were orally administered l-glutamine (1.92 g/kg) for 13 days (**Figure 1**). Meanwhile, mice in groups A and B were treated with an equivalent volume of distilled water. Except for the mice in the normal control group, all other mice received intraperitoneal injections of 5-FU (50 mg/kg) from day 6 to day 10 to induce an experimental intestinal mucositis model according to our preliminary experimental results, which was similar to Yasuda M's report (Yasuda et al., 2012). At the end of the treatment, the mice were sacrificed by cervical dislocation under mild anesthesia. Blood and small intestine samples were collected for further analysis.

### Body Weight and Diarrhea Assessment

As a marker of the general health of the mice, the body weight was recorded daily during the experiment and the percent of initial body weight was utilized to evaluate the weight gain. After the first injection of 5-FU, the diarrhea of each animal was assessed every day by two well-trained researchers blinded to the treatment allocation. The severity of diarrhea was recorded by a diarrhea score that was described by Kurita A (Kurita et al., 2000) as follows: 0, normal (normal stool or absent); 1, slight (slightly wet and soft stool); 2, moderate (wet and unformed stool with moderate perianal staining of the coat); and 3, severe (watery stool with severe perianal staining of the coat). The average diarrhea score was used to evaluate the severity of diarrhea.

### Intestinal Histology and Morphometry

To evaluate intestinal mucositis following the injections of 5-FU, a 2-cm segment of distal ileum was surgically collected, washed with PBS and fixed in 4% paraformaldehyde. The specimens were then dehydrated and embedded in paraffin wax. Then, approximately 5-μm-thick sections were cut and routinely stained with hematoxylin and eosin (H&E) for histopathological observation. Images were captured by an LEICA DM1000 microscope (LEICA, Germany) (magnification, 200×). For each H&E section of ileum tissue, the average villus height and crypt depth were measured for 10 random villi and crypts on each slide with Image J software.

### Analyses of Intestinal Injury Markers

The serum levels of d-lactic acid, DAO, and endotoxin were examined as markers for intestinal injury. At the end of this experiment, blood samples were collected from the orbital vein. Serum was prepared by the centrifugation of the blood samples at 3500 g (Sorvall ST-16R, Thermo) for 10 min at 4°C. Enzyme-linked immunosorbent assay (ELISA) kits were utilized to measure the serum levels of d-lactic acid, DAO, and endotoxin in accordance with the protocols provided by the manufacturers.

### Immunohistochemical Analysis

Indirect immunohistochemical analysis was carried out to evaluate the expression of occludin, claudin-1, and ZO-1. Briefly, approximately 5-μm-thick ileum sections were fixed with 4% paraformaldehyde, dehydrated, and paraffin-embedded. These sections were then deparaffinized in xylene and rehydrated with graded ethanol. Then, antigen retrieval was performed by microwaving the sections for 20 min in 0.01 M citrate acid buffer (pH 6.0). Next, to block endogenous peroxidase activity, the sections were incubated in 3% hydrogen peroxide for 10 min, followed by a PBS wash. Afterwards, the sections were incubated with normal goat serum or 30 min at 37°C, followed by an overnight incubation with primary antibodies against occludin, claudin-1, or ZO-1 at 4°C and an incubation with the secondary antibody (goat anti-rabbit IgG-HRP) for 60 min at 37°C. Negative controls were prepared by replacing the primary antibodies with PBS solution. Then, the sections were stained with a diaminobenzidine kit and were counterstained in hematoxylin. Occludin, claudin-1, or ZO-1 expression was examined by the brown-colored immunohistochemical staining, which was imaged by a light microscope at a magnification of 200×. Image-Pro Plus software was utilized to analyze the staining intensity as the average optical density (AOD). All the analyses were performed in a blinded manner by the same experienced investigator.

### Statistical Analyses

All the data presented are expressed as the mean ± the standard error of the mean (SEM). Two-way univariate analysis of variance (ANOVA) with repeated measures on time tested the body weight, using different treatment as a between-subject factor, time intervals as a within-subject factor, and body weight gain as a dependent variable. Bonferroni's *post hoc* test was used to compare the body-weight gain of mice in model group to that of other groups. Statistical differences of all the continuous variable between groups were analyzed *via* one-way ANOVA. Non-parametric test was used to compare the diarrhea score. Differences of *P* < 0.05 were considered statistically significant (marked as ^*^). A higher significance level was set at *P* < 0.01 (marked as ^**^).

## Results

### Identification of the Characteristic Components in G-SJZ

**Figure 2:**
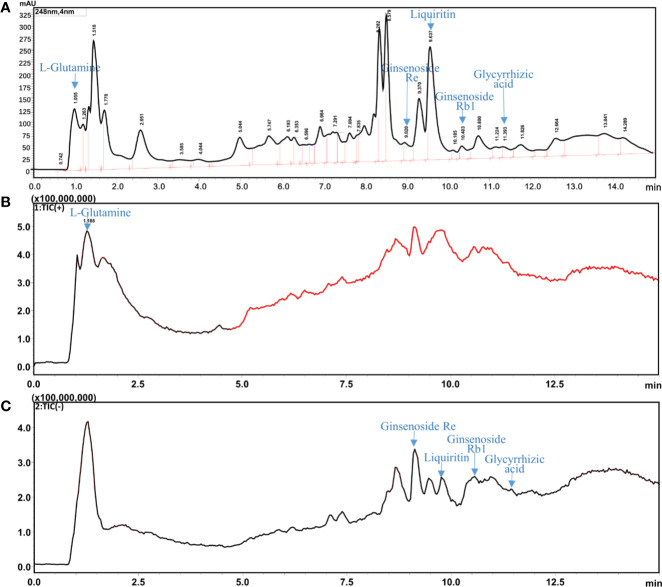
UHPLC-ESI-MS/MS analysis of G-SJZ: HPLC chromatogram with UV wavelength at 248 nm **(A)**, total ion chromatogram (TIC) of G-SJZ in positive ion mode **(B)** and negative ion mode **(C)**.

UHPLC-ESI-MS/MS analysis of the characteristic chemical composition of the combination was performed. l-glutamine, the standard compound, was analyzed by HPLC with UV at 235 nm and found to be at 1.055 min. The total ion chromatogram in **Figure 2B** indicated its retention time was 1.188 min. The mass spectrum was shown in the **Supplementary Figure 1**. In addition, l-glutamine was successfully identified in G-SJZ water extract solution. Furthermore, in accordance with the Chinese Pharmacopoeia (2015 edition), there are four main components including ginsenoside Re, ginsenoside Rb1, liquiritin, and glycyrrhizic acid as the components for quality control that presenting at *Ginseng* radix et rhizome and *Glycyrrhizae* radix et rhizome were also identified at 9.020, 10.403, 9.637, and 11.393 min, respectively (**Figure 2** and **Supplementary Figure 2**). Taken together, these data suggested that the main bioactive components could be identified in G-STZ, and which provided a reliable and scientific support for the following activity evaluations *in vivo*.

### Effect of G-SJZ on Body-Weight Loss During the Experiment

**Figure 3:**
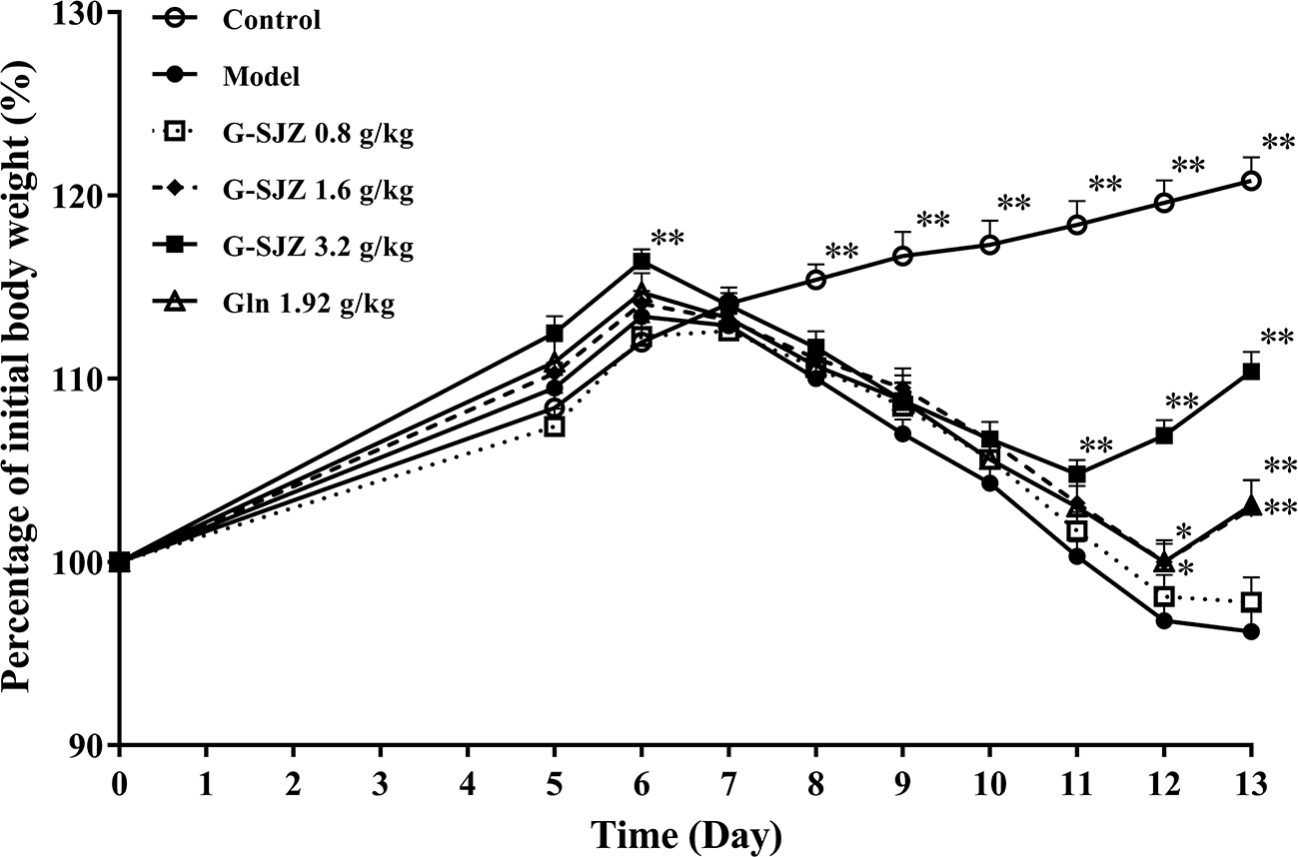
Effect of G-SJZ on body-weight loss in mice following the continuous administration of 5-Fu. ^*^Significant difference (*P* < 0.05) and ^**^P < 0.01 versus the model group. G-SJZ, combination composition composed of glutamine and Si-Jun-Zi-Tang; Gln, glutamine.

The body weight of the mice was observed throughout the experiment (**Figure 3**). Before the administration of 5-Fu, the body weight of the mice in the control and model group exhibited normal increases. After the injection of 5-Fu, mice gradually lost weight, whereas normal increases were still seen in the control group. Compared to the control group, in the model group, a marked decrease was observed since the third injection of 5-Fu on day 8 (*P* < 0.01) and lasted until the end of the experiment, falling to the lowest point on day 13.

Two-way ANOVA with repeated measures on time revealed that different treatment influenced the body-weight gain significantly [*F* (5, 63) = 15.686, *P* = 0.001], in which the Bonferroni's *post hoc* test indicated that G-SJZ at 3.2 g/kg significantly enhance the body-weight gain (*P* = 0.009). Besides, two-way ANOVA with repeated measures on time indicated a significant effect of time intervals on the body-weight gain [*F* (8, 504) = 257.810, *P* = 0.001]. Significant interactions were observed as well [*F*(40, 504) = 41.985, *P* = 0.001].

When tested by one-way ANOVA, although the body weight of G-SJZ mice was still lower than that of normal mice (*P* < 0.01), ongoing treatment with G-SJZ resulted in a larger body-weight gain following continuous injections with 5-Fu than that in the model group mice, with significant differences since day 11 in the 3.2 g/kg G-SJZ group (*P* < 0.01) and since day 12 in the 1.6 g/kg G-SJZ group (*P* < 0.05, *P* < 0.01). Meanwhile, mice in the 1.92 g/kg l-glutamine group exhibited a significant increase in weight gain on day 12 and day 13 of this experiment (*P* < 0.05, *P* < 0.01). Moreover, mice pre-treated with 3.2 g/kg G-SJZ, containing 1.92 g of l-glutamine and 5.12 g of the raw herbs included in Si-Jun-Zi-Tang, got significantly more body-weight gain than mice pre-treated with only 1.92 g/kg l-glutamine on day 12 and day 13 (*P* < 0.01).

### Effect of G-SJZ on the Severity of Diarrhea

**Figure 4:**
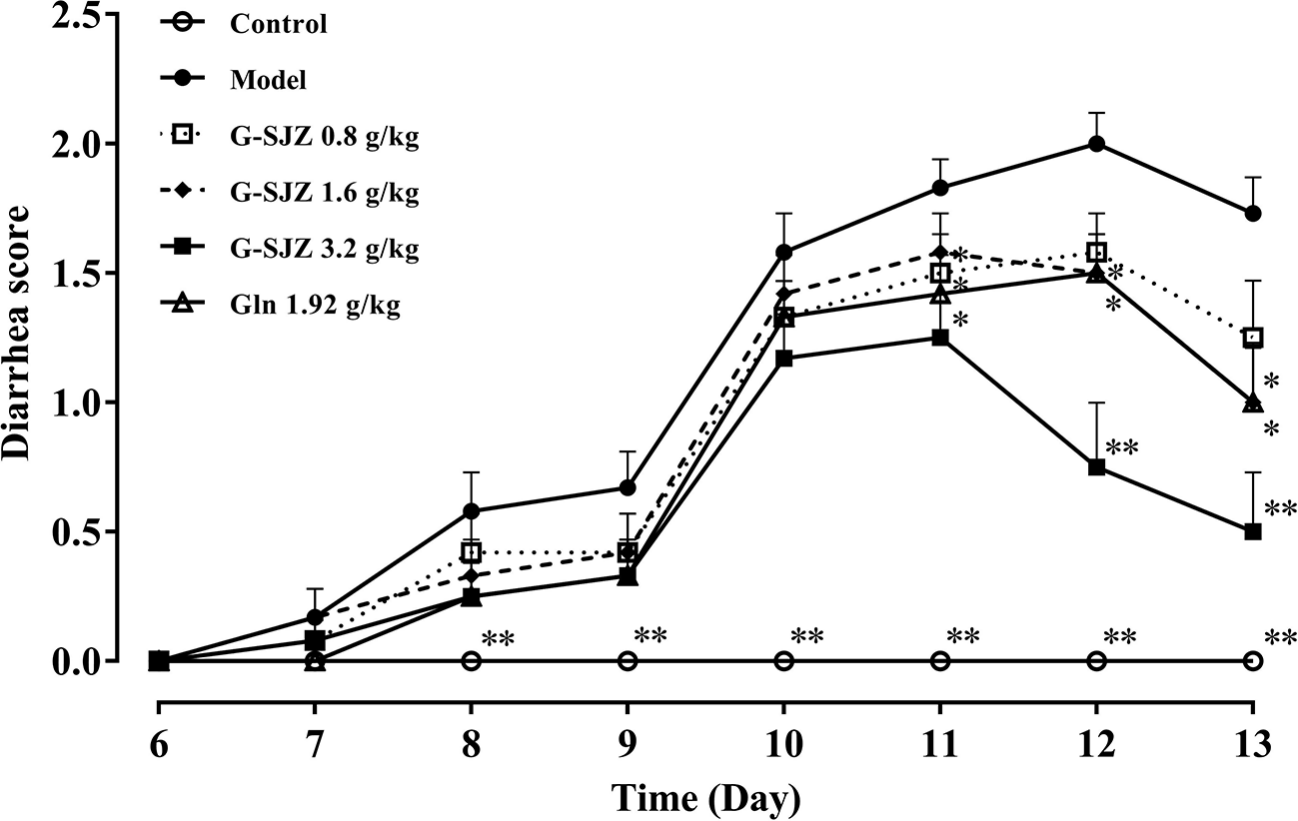
Effect of G-SJZ on the severity of diarrhea in mice following consecutive injections of 5-Fu. ^*^Significant difference (*P* < 0.05) and ^**^P < 0.01 versus the model group. G-SJZ, combination composition composed of glutamine and Si-Jun-Zi-Tang; Gln, glutamine.

Intestinal mucositis is usually accompanied by diarrhea in the clinic. As expected, the consecutive administration of 50 mg/kg 5-Fu led to severe diarrhea in mice. As shown in **Figure 4**, mice in the model group suffered from diarrhea since the third injection of 5-Fu on day 8, and the diarrhea score was the highest on day 12 (*P* < 0.01). After pre-treatment with G-SJZ and l-glutamine, there was a 1- or 2-day delay in the onset of this diarrhea status in mice exposed to repeated injections of 5-Fu. Since day 11, mice in the G-SJZ (1.6 and 3.2 g/kg) and l-glutamine (1.92 g/kg) groups had lower diarrhea scores than mice in the model group (*P* < 0.05, *P* < 0.01), with mice in only the 3.2 g/kg G-SJZ-treated group showing no significant difference compared with mice in the control group on day 13. Moreover, mice in the 3.2 g/kg G-SJZ group had a lower increase in the diarrhea score on day 12 and day 13 when compared to the 1.92 g/kg l-glutamine group (*P* < 0.05).

### Effect of G-SJZ on Histological Changes in the Intestinal Mucosa

**Figure 5:**
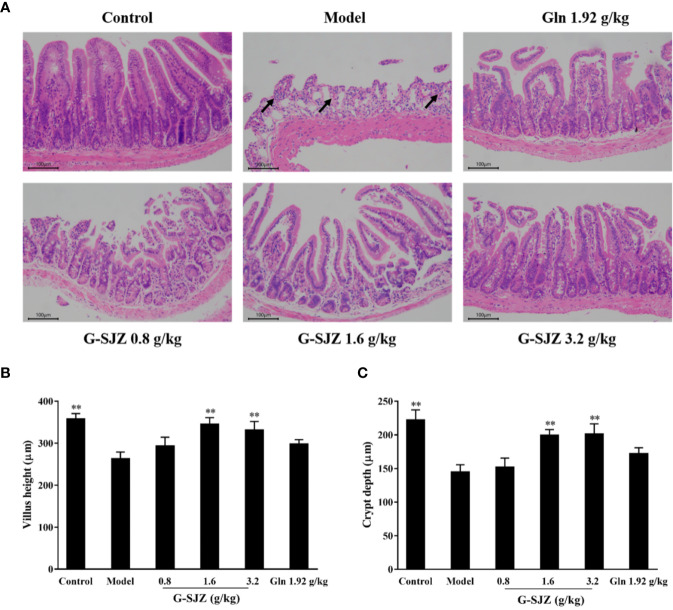
Effect of G-SJZ on histological changes in the intestinal mucosa of mice administered 5-Fu: representative images of paraffin sections of the ileum stained with H&E **(A)** in which mice in model group showed goblet cell depletion, the damage or loss of villus and crypt structures, vacuolization and intense inflammatory infiltrate (arrows), while pre-treatment with G-SJZ (1.6 and 3.2 g/kg) or Gln had a high tendency to alleviate these changes; lengths of the villi **(B)** and depths of the crypt **(C)**. ^*^Significant difference (*P* < 0.05) and ^**^P < 0.01 versus the model group. G-SJZ, combination composition composed of glutamine and Si-Jun-Zi-Tang; Gln, glutamine.

As shown in **Figure 5A**, the ileum sections of mice after consecutive injections of 50 mg/kg 5-Fu presented histologic changes including shedding or atrophied villi, goblet cell depletion, the damage or loss of villus and crypt structures, vacuolization, and intense inflammatory infiltrate. The morphometry showed that the villi length as well as the crypt depth of mice in the model group was markedly decreased compared to those in the mice in the control group (*P* < 0.01) (**Figures 5B, C**). In contrast, pre-treatment with G-SJZ (1.6 and 3.2 g/kg) and l-glutamine (1.92 g/kg) had a high tendency to alleviate the histopathologic changes compared to mice in the model group. Additionally, pre-treatment with G-SJZ (1.6 and 3.2 g/kg) reversed the decrease of villi length and crypt depth (*P* < 0.01). However, no significant difference was observed between the 1.92 g/kg l-glutamine and model groups (*P* > 0.05).

### Effect of G-SJZ on Intestinal Injury Markers

**Figure 6:**
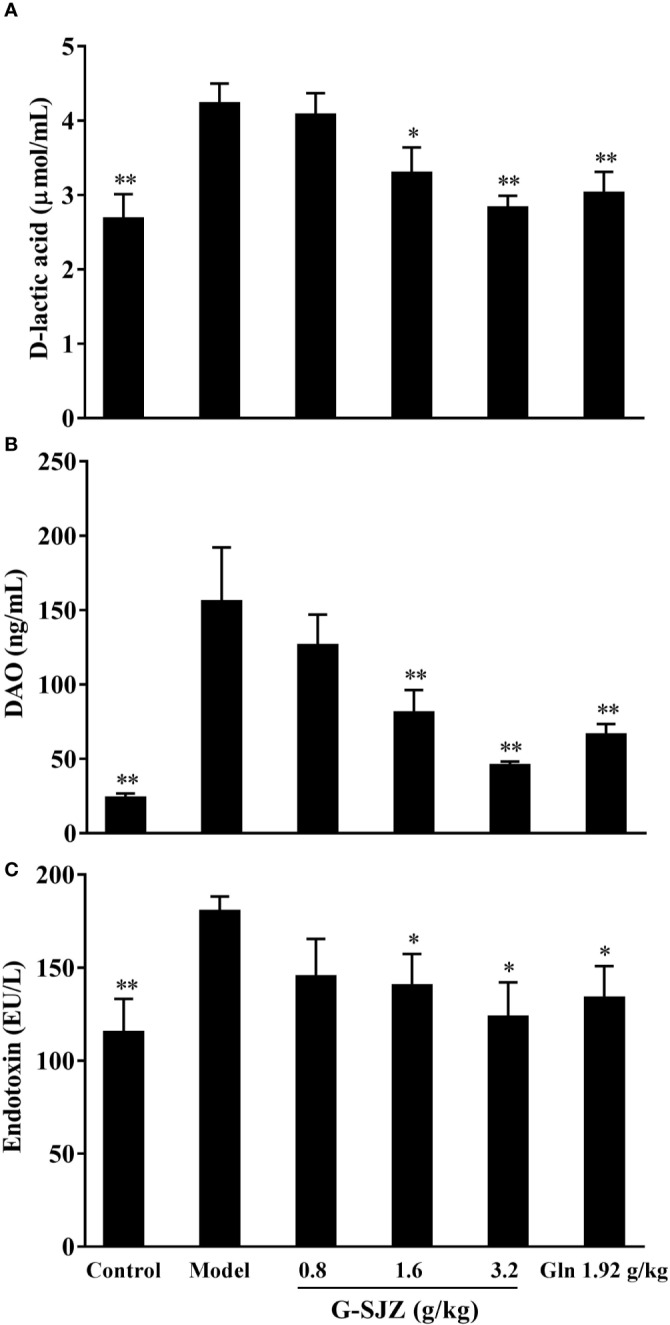
Effect of G-SJZ on serum intestinal injury markers in mice administered continuous 5-Fu: D-lactic acid **(A)**, DAO **(B)** and endotoxin **(C)**. ^*^Significant difference (*P* < 0.05) and ^**^P < 0.01 versus the model group. G-SJZ, combination composition composed of glutamine and Si-Jun-Zi-Tang; Gln, glutamine.

In addition to examining severe diarrhea and histological damage, we detected the serum level of d-lactic acid, DAO, and endotoxin to examine the intestinal injury resulting from the constant exposure of mice to 5-Fu. As shown in **Figure 6**, injections of 5-Fu caused a significant increase in the serum levels of these three injury markers compared with the control treatment (*P* < 0.01). Accordingly, the levels of serum d-lactic acid, DAO, and endotoxin significantly decreased in the G-SJZ (1.6 and 3.2 g/kg) and l-glutamine (1.92 g/kg) groups (*P* < 0.05, *P* < 0.01) compared to those in the model group. Furthermore, although there were no significant differences between the G-SJZ (3.2 g/kg) and l-glutamine (1.92 g/kg) groups, a trend of lower levels of DAO and endotoxin was observed in the former group.

### Effect of G-SJZ on the Immunohistochemical Detection of Occludin, Claudin-1, and ZO-1

**Figure 7:**
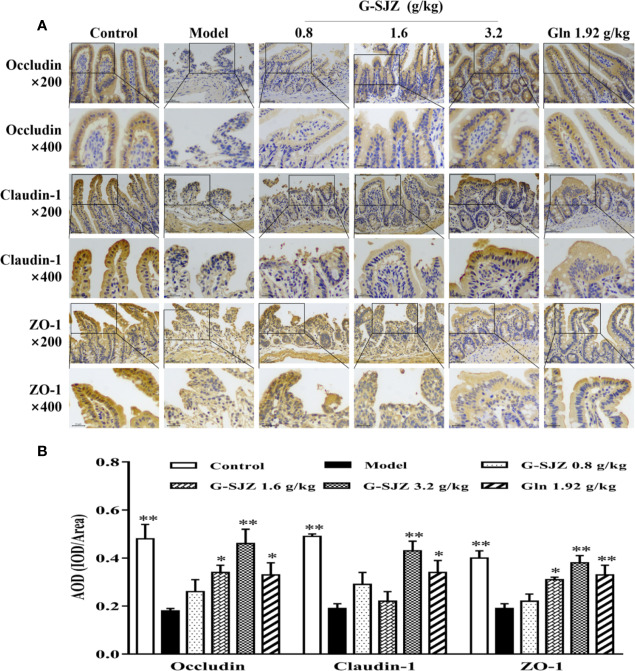
Effect of G-SJZ on the expression of occludin, claudin-1, and ZO-1 in mice administered continuous 5-Fu: representative images of immunohistochemical staining under a microscope of 200 and 400 magnifications **(A)**, AOD analyzed by Image-Pro Plus **(B)**. ^*^Significant difference (*P* < 0.05) and ^**^P < 0.01 versus the model group. G-SJZ, combination composition composed of glutamine and Si-Jun-Zi-Tang; Gln, glutamine.

As suggested above, continuous injections of 5-Fu damaged the intestinal mucosa and elevated the circulating intestinal injury markers in mice. Thus, 5-Fu may affect the tight junction which contributes to the function of the physical intestinal barrier and consists of two functional protein categories, integral transmembrane proteins that form a network between adjacent cell membranes and peripheral membrane adaptor proteins. Occludin and claudin-1 are the main cytoplasmic transmembrane proteins, while ZO-1 is the most important cytoplasmic adaptor protein (Lee et al., 2018). Therefore, the immunohistochemical expression of occludin, claudin-1, and ZO-1 was determined in ileum sections. As shown in **Figure 7**, mice exposed to continuous 5-Fu exhibited a dramatic decrease in the expression of occludin, claudin-1, and ZO-1 compared with mice in the control group (*P* < 0.01). On the other hand, mice in the G-SJZ (1.6, 3.2 g/kg) and l-glutamine (1.92 g/kg) groups had significantly higher expressions of these proteins than the model mice (*P* < 0.05, *P* < 0.01), with the 3.2 g/kg G-SJZ group exhibiting the highest value, which was extremely close to the value in normal mice.

## Discussion

Due to the important physiological roles of the gastrointestinal system, intestinal mucositis caused by anti-neoplastic therapies is debilitating, sometimes potentially life threatening, and is likely to lower patients' compliance, which usually gives rise to an alteration or interruption in patients' treatment regimen; thus, intestinal mucositis not only has a negative impact on the therapeutic outcomes and quality of life of patients but also increases the cost of treatment (Ribeiro et al., 2016). Thus, recovery from this toxicity as soon as possible will enormously benefit patients, health care providers, and payers. Unfortunately, the prevention and treatment of intestinal mucositis has received little attention. In the MASCC/ISOO guidelines, based on the evidence level, only probiotics were suggested for the prevention of cancer treatment-related intestinal mucositis, and no therapeutic agents could be strongly recommended at present (Bowen et al., 2019). Intestinal mucositis induced by cancer treatment has been an extremely challenging task in clinical practice for a long time, mainly owing to there being little progress in the development of potential new drugs, which has been largely due to the complicated pathophysiology of intestinal mucositis. Based on developments in systems biology, multiple components and multiple targets are increasingly believed to be more effective than single compounds, particularly for complicated disorders (Fu et al., 2014). To deal with intestinal mucositis, multidisciplinary and systematic thinking are likely to be required.

l-glutamine has been reported to ameliorate mucositis induced by radiotherapy and chemotherapy and has gained wide attention since the late 20th century (Skubitz and Anderson, 1996). Thereafter, inconstant results were obtained, which indicated that l-glutamine failed to affect the occurrence of doxifluridine-induced diarrhea or the severity of stomatitis, nausea, and diarrhea caused by 5-fluorouracil (5-FU)/calcium-folinate (CF) chemotherapy (Bozzetti et al., 1997; Decker-Baumann et al., 1999; Alpers, 2006). A meta-analysis showed that glutamine could reduce the duration of chemotherapy induced diarrhea in patients but could not improve its severity (Sun et al., 2012). In contrast, rats bearing the Ward colon tumor pretreated with oral glutamine showed less body weight loss and severity of diarrhea after trinotecan treatment (Xue et al., 2007). In the present study, we observed similar effect of l-glutamine on the body weight and diarrhea of mice undergoing continuous injections with 50 mg/kg 5-Fu with the research in rats (Xue et al., 2007). Moreover, pre-treatment with G-SJZ, which is composed of l-glutamine and the traditional Chinese herbal formula Si-Jun-Zi-Tang, showed better potential than l-glutamine alone at the same dose in prevention of body-weight loss and severe diarrhea, regarding to an earlier and stronger reversal effect on both weight loss and the severity (or onset) of the diarrhea status (**Figures 3** and **4**). Si-Jun-Zi-Tang is a traditional herbal formula that is used in gastroenterology and is also frequently utilized as a complementary agent for oncologic treatment due to its multiple roles in preventing tumor recurrence and metastasis and improving the quality of life (Lu et al., 2018; Zhou et al., 2019). Although there might be specific differences between human and rodents, we speculate that Si-Jun-Zi-Tang might compensate for the deficiencies of l-glutamine in addressing the physical manifestations of intestinal mucositis following anticancer drug treatment considering its multiple components and multiple targeted effects and the clinical practice, which possibly resulted in the higher efficacy of G-SJZ in the current study.

As for the microscopic presentations, 5-Fu has been shown to destroy the integrity of the mucosa, to reduce the villi length and crypt depth and to increase the severity of inflammation, thus leading to the macroscopic symptoms of nausea, diarrhea, anorexia, and weight loss (Zhang et al., 2018). The H&E staining (**Figure 5**) in this study showed that G-SJZ attenuated the histopathological and morphologic changes induced by 5-Fu and was more effective than l-glutamine alone, especially in the restoration of the villi length and crypt depth. It can be seen that the 1.92 mg/kg dose of l-glutamine did not markedly reversed effects as the 1.6 g/kg (including 0.96 g/kg of l-glutamine) and 3.2 g/kg (including 1.92 g/kg of l-glutamine) doses of G-SJZ did. The failure of l-glutamine alone in morphometry in our study was similar to Carneiro-Filho BA's finding that oral l-glutamine was not able to ameliorate some of the morphologic effects, including the villus height, in mice treated with 5-Fu (Carneiro-Filho et al., 2004). The author also presumed this was mainly because of its poor chemical stability and weak aqueous solubility; thus, the superior efficacy observed in G-SJZ (even at a lower dose of 1.6 g/kg G-SJZ) might be partially ascribed to the improvement of these characteristics of l-glutamine *via* the multiple targeted effects of Si-Jun-Zi-Tang. In addition, the increase in circulating intestinal injury markers after injections of 5-Fu was considered a result of heightened permeability, which was presumably caused by the destruction of the integrity of the intestinal mucosal that was seen in histopathological analyses. Daniele B reported that l-glutamine had a protective effect on 5-Fu-induced diarrhea, which was largely associated with reductions in permeability (Daniele et al., 2001). Our findings also indicated that l-glutamine had a similar effect, and l-glutamine may significantly decrease the serum levels of d-lactic acid, DAO, and endotoxin. Moreover, although the effects were not as pronounced as it was in the H&E staining, pre-treatment with G-SJZ also caused a trend of a stronger restoration of circulating DAO and endotoxin than l-glutamine alone (**Figure 6**). The action of G-SJZ based on the microscopic presentations was homologous to that observed in the macroscopic manifestations.

Changes in tight junction proteins are strongly related to heightened permeability after chemotherapy (Hamada et al., 2010). Beutheu S described the regulatory role of occludin and claudin-1 by which l-glutamine prevented gut barrier disruption, as shown by improved intestinal permeability in a rat model of intestinal mucositis induced by methotrexate (Beutheu et al., 2014). When l-glutamine was combined with arginine, which may limit the modification of occludin and ZO-1 expression in methotrexate-treated Caco-2 cells (Beutheu et al., 2013), the same effect was not observed. In the current investigation, both l-glutamine alone and l-glutamine combined with Si-Jun-Zi-Tang showed a similar effect on the regulation of occludin, claudin-1, and ZO-1, indicating that there were possibly no significant changes on this parameter when l-glutamine was combined with Si-Jun-Zi-Tang. Thus, the superior protective effect of the combined composition G-SJZ on intestinal mucositis might not be attributed to additional alterations in tight junction proteins originating from the addition of the herbal formula.

Additionally, a preliminary result of bacterial translocation, as determined by a method in which homogenized spleen is cultured with aerobic and anaerobic media, was a marked increase in the growth of bacteria after repeated injections of 5-Fu. This effect was associated with the injury of the intestinal mucosa and was similar to Yang's finding on another anticancer drug, cyclophosphamide (Yang et al., 2013). Their results showed that cyclophosphamide potentially increased pathogenic bacteria count in rats and indicated that the regulation of the mechanical barrier and bacterial colonization might represent a hopeful direction for intestinal mucositis. We observed a less extensive restoration of the increased positive growth of bacteria resulting from 5-Fu in mice pre-treated with l-glutamine alone than in mice pre-treated with G-SJZ (**Supplemental Figure 3**). Moreover, a previous study showed that one of the major effective components in Si-Jun-Zi-Tang, a polysaccharide, was able to regulate the abundance of intestinal bacterial genera (Gao et al., 2018). Therefore, we speculate that the modulation of colonization might partially contribute the stronger ability of G-SJZ to ameliorate the intestinal mucositis induced by 5-Fu. However, this is only a hypothesis and needs further investigation for confirmation.

## Conclusion

In summary, G-SJZ, a combination composition composed of l-glutamine and the traditional Chinese herbal formula Si-Jun-Zi-Tang, had a protective effect against intestinal mucositis that was caused by the constant exposure of mice to 5-Fu. Moreover, G-SJZ showed a tendency of stronger potency than the same dose of l-glutamine alone. As Si-Jun-Zi-Tan has multiple components and multiple targeted effects, we speculate that the Si-Jun-Zi-Tang in G-SJZ may compensate for the deficiencies of l-glutamine in this model. According to our results, this compensation might not be associated with an additional modulation in tight junction proteins resulting from the addition of the herbal formula to l-glutamine, and the mechanism still needs further study. Nevertheless, our findings provide important data to support clinical studies on G-SJZ in 5-Fu-induced intestinal mucositis, and for the first time suggest that the combined use of l-glutamine and Si-Jun-Zi-Tang might be more effective than l-glutamine alone. G-SJZ may become a preferable choice for cancer patients with intestinal mucositis, as there are limited choices in the clinic.

## Data Availability Statement

All datasets generated for this study are included in the article/**Supplementary Material**.

## Ethics Statement

The animal study was reviewed and approved by the animal ethics committee of Chengdu University of Traditional Chinese Medicine.

## Author Contributions

LQ designed this study, interpreted the data and wrote this manuscript. WT conducted the research and analyzed the data. YJ contributed to the UHPLC-MS analysis of G-SJZ. LL contributed to the animal experiments. SL contributed to the histological and immunohistochemical analysis. WZ and JW funded the study and approved the submission of this manuscript. All authors contributed to the article and approved the submitted version.

## Funding

This work was supported by Innovation Fund for Young Teachers (No. ZRQN1769) and Xinglin Scholar Research Promotion Project (No. BSH2018018) of Chengdu University of Traditional Chinese Medicine.

## Conflict of Interest

The authors declare that the research was conducted in the absence of any commercial or financial relationships that could be construed as a potential conflict of interest.
