# Supplementary material for: Combination Compositions Composed of l-Glutamine and Si-Jun-Zi-Tang Might Be a Preferable Choice for 5-Fluorouracil-Induced Intestinal Mucositis: An Exploration in a Mouse Model
Source: Front Pharmacol. 2020 Jun 17;11:918. doi: 10.3389/fphar.2020.00918 (PMC7313676; doi:10.3389/fphar.2020.00918)
Supplement: Supplementary file 1 [file DataSheet_1.docx]

## Supplementary Figures


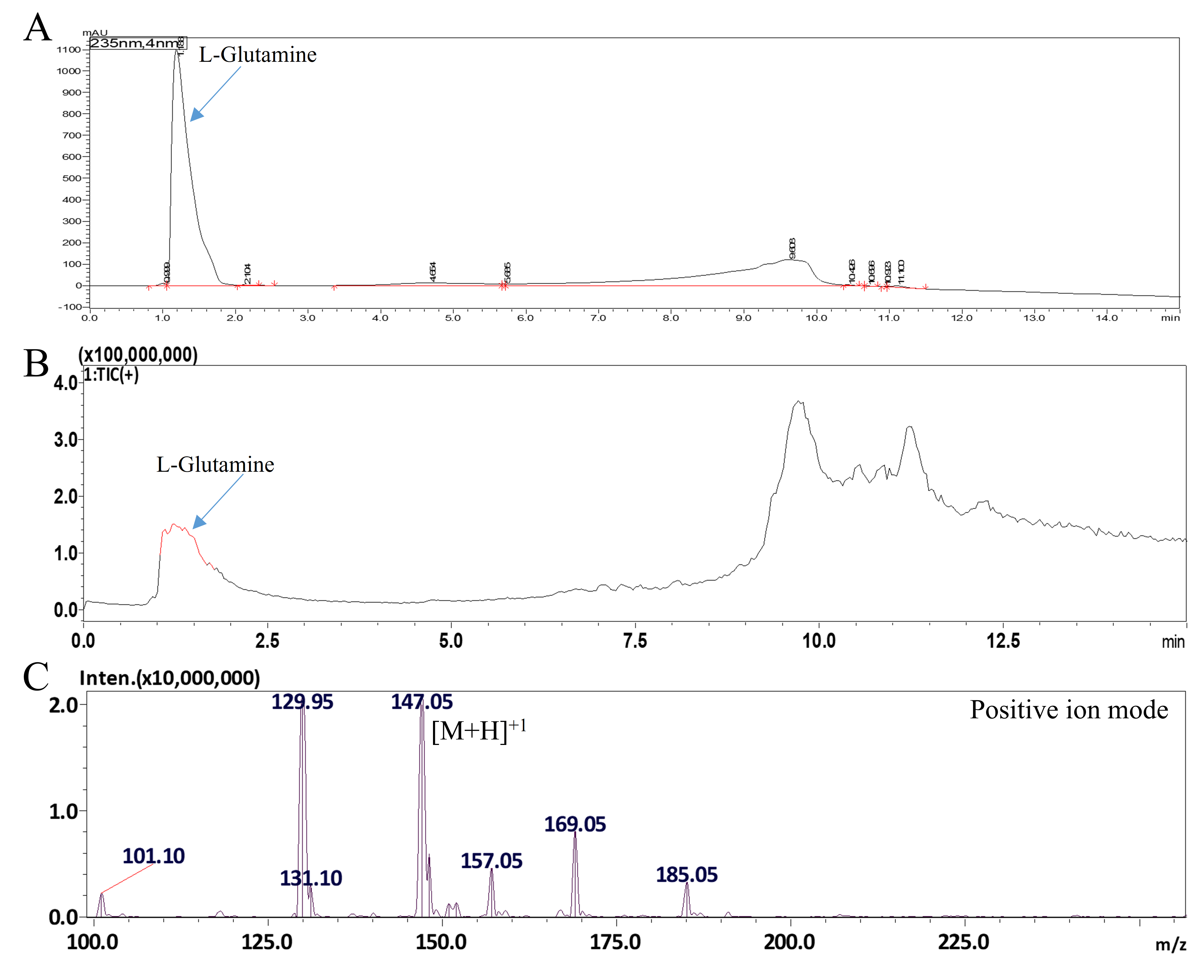


**Supplementary Figure 1.** UHPLC-ESI-MS/MS analysis of L-Glutamine: HPLC with UV wavelength at 235 nm (A), total ion chromatogram (TIC) of L-Glutamine in positive ion mode (B) and its mass spectrum (C).


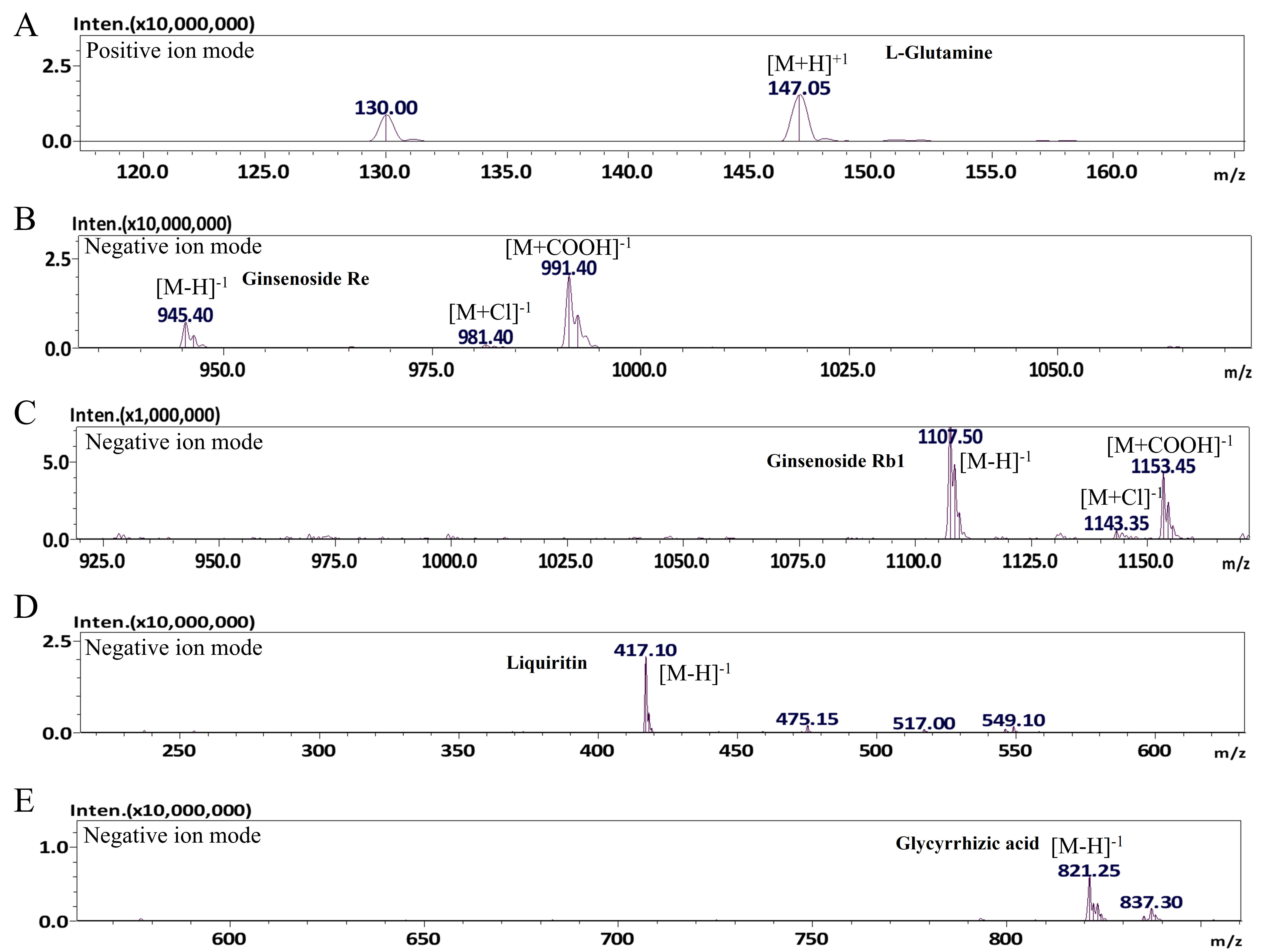


**Supplementary Figure 2.** Mass spectrum of L-Glutamine (A), ginsenoside Re (B), ginsenoside Rb1 (C), liquiritin (D) and glycyrrhizic acid (E).


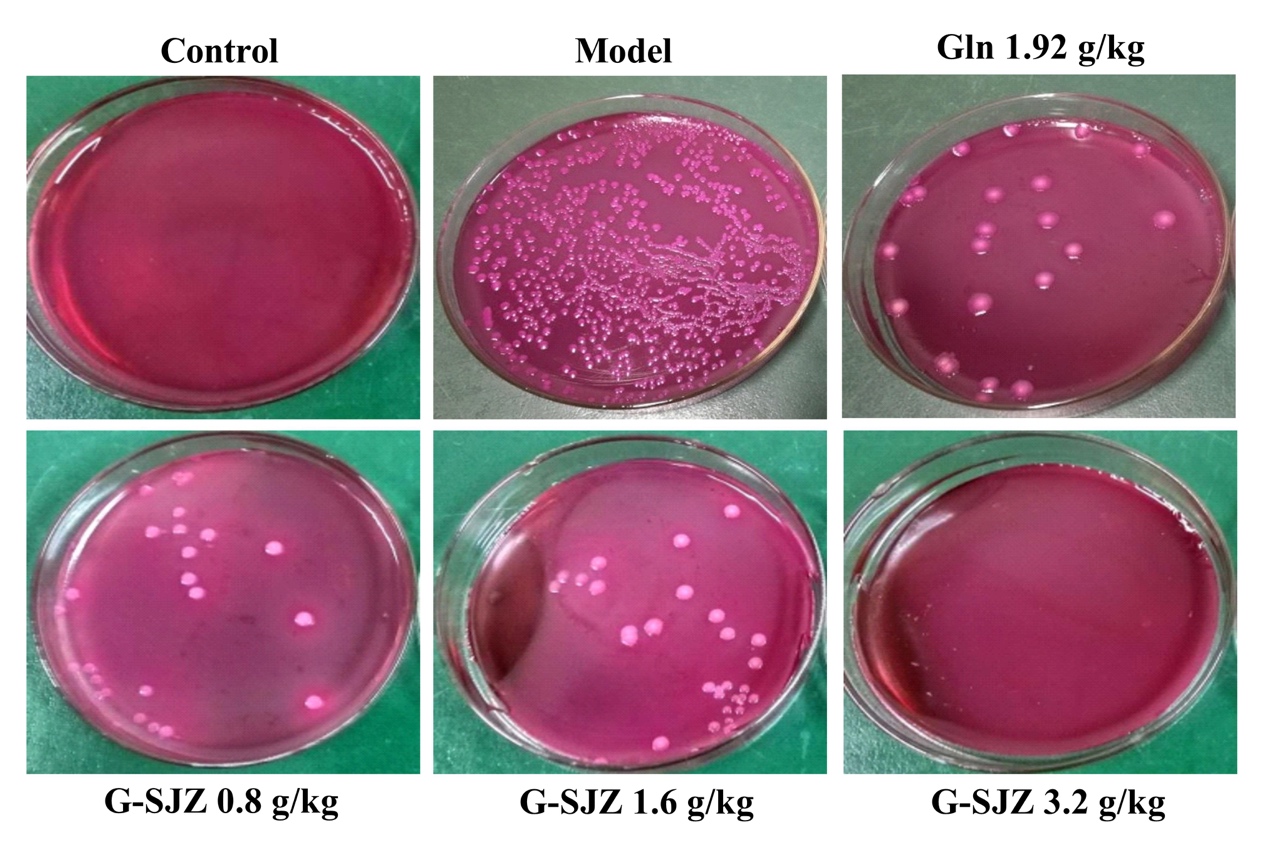


**Supplementary Figure 3.** Bacterial colonies of homogenized spleen cultured with MacConkay agar. G-SJZ, combination composition composed of glutamine and Si-Jun-Zi-Tang; Gln, glutamine.
